# Violence at School and Bullying in School Environments in Peru: Analysis of a Virtual Platform

**DOI:** 10.3389/fpsyg.2020.543991

**Published:** 2021-01-13

**Authors:** Wendy Arhuis-Inca, Miguel Ipanaqué-Zapata, Janina Bazalar-Palacios, Nancy Quevedo-Calderón, Jorge Gaete

**Affiliations:** ^1^Instituto de Investigación, Universidad Católica Los Ángeles de Chimbote, Chimbote, Peru; ^2^Facultad de Educación y Humanidades, Escuela Profesional de Educación, Universidad Católica Los Ángeles de Chimbote, Satipo, Peru; ^3^Faculty of Education, Universidad de los Andes, Santiago, Chile; ^4^ANID, Millennium Science Initiative Program, Millennium Nucleus to Improve the Mental Health of Adolescents and Youths, Imhay, Santiago, Chile

**Keywords:** school violence, bullying, aggressor, school environment, Peru

## Abstract

**Background:**

School violence and bullying are prevalent problems that affect health in general, especially through the development of emotional and behavioral problems, and can result in the deterioration of the academic performance of the student victim. The objective of this study was to determine the prevalence rates of aggressive behaviors according to types of school violence and bullying, sociodemographic characteristics, and variation by department, region, and time in the period between 2014 and 2018 in Peru.

**Methods:**

The design was observational and cross-sectional based on data from the Specialized System for Reporting Cases of School Violence (Sistema Especializado en Reporte de Casos sobre Violencia Escolar—SíseVe) in Peru, which covers a population of 23,641 students at the initial, primary, and secondary levels of Basic Regular Education [Educación Básica Regular (EBR)], for the 2014–2018 period. The prevalence rates of the different types of school violence and bullying, the sociodemographic characteristics, and the variation by department, region, and time in the period between 2014 and 2018 were estimated.

**Results:**

Psychological violence/bullying occurred at higher prevalence rates (185.8 and 62.6 per 100,000 residents). Women from public institutions reported greater sexual violence, mostly by teachers (67.8%) than by other students (32.2%). The Selva region had the highest prevalence rate of sexual violence (10.1 per 100,000 residents). The departments of Tacna and Piura had the highest and lowest rates of psychological/verbal violence and bullying in 2018 (95.79 and 25.31 per 100,000 residents).

**Conclusion:**

Psychological/verbal violence and bullying is highly prevalent among students; women report being victims of sexual violence by administrative personnel of public institutions. The Selva region had the highest rate of sexual violence, and Piura and Tacna had the highest and lowest rates of violence and psychological/verbal bullying. Based on these results, it is suggested to conduct evidence-based prevention programs in Peruvian schools to reduce these social problems.

## Introduction

School violence is any type of physical, psychological, or verbal and/or sexual aggression among students, teachers, and/or school personnel toward a student (United Nations Educational Scientific and Cultural Organization [UNESCO], 2019). On the other hand, bullying is defined as deliberate and repetitive aggressive behavior over time, accompanied by an imbalance of power between the aggressor or aggressors and the victim. Bullying occurs only among students and can be categorized into four types: physical, psychological/verbal, sexual, and cyberbullying (United Nations Educational Scientific and Cultural Organization [UNESCO], 2019). These phenomena are still predominant problems in the school population and are associated with the development of emotional and behavioral problems among victims. In addition, they impact mental and physical health, and academic performance of victims (Hidalgo-Rasmussen et al., 2018; Jackson et al., 2019; Koyanagi et al., 2019; United Nations Educational Scientific and Cultural Organization [UNESCO], 2019).

Worldwide, 246 million children and adolescents each year are victims of any type of school violence or bullying (United Nations Educational Scientific and Cultural Organization [UNESCO], 2017). In relation to school violence, more than one-third of students have been physically attacked by their peers. On the other hand, the same study shows that 47.2% of 144 countries still allow physical punishment of school children by teachers in schools (United Nations Educational Scientific and Cultural Organization [UNESCO], 2019). As for bullying, 32% of schoolchildren are victims of this social problem, the most frequent type being psychological/verbal (United Nations Educational Scientific and Cultural Organization [UNESCO], 2019). In relation to cyberbullying, we find a high variability, where on the one hand we have studies carried out in the United States and the United Kingdom that show a prevalence between 5 and 20% (Sourander et al., 2010); while other countries such as Denmark and Romania report prevalences of 41.0 and 25.0%, respectively (Mascheroni and Cuman, 2014). Prevalences of cyberbullying comparing what happens in primary vs. secondary education also show high variability. For example, in France, we see that 14% of primary school students reported being victims of cyberbullying, while only 5% of secondary school students reported the same (Catherine and Michael, 2016). Other studies show that cyberbullying victimization decreases in prevalence as children grow older (Ševčíková and Šmahel, 2009; Wang et al., 2019). This variability may be explained by cultural differences and the level of access to technologies that students in different countries have.

One of the most dramatic manifestations of school violence is related to sexual violence or abuse. A UNESCO report, which only included Central African countries, reported that 7.1% of women at age 15 were victims of sexual violence by their teachers. For example, in Liberia, one of the poorest countries in Africa, a high proportion of girls were found to be victims of sexual violence perpetrated by teachers and school religious personnel (Steiner et al., 2018). The same United Nations Educational Scientific and Cultural Organization [UNESCO] (2019) report indicates that there are little data and evidence of sexual violence perpetrated by peers or physical or sexual violence perpetrated by teachers in other regions, such as Latin America (Contreras et al., 2010; Eljach, 2011).

In relation to factors associated with school violence, it has been seen that physical violence appears to be more frequent among men either in the case of peers (57.3%) or school staff (33%) (Romaní and Gutiérrez, 2010; Miranda, 2016). On the other hand, psychological violence seems to be more frequent in women (United Nations Educational Scientific and Cultural Organization [UNESCO], 2019). Physical bullying is more prevalent among male students (García et al., 2010; United Nations Educational Scientific and Cultural Organization [UNESCO], 2019). On the other hand, there are studies that show a higher prevalence of school violence in public schools compared to private ones (Romaní and Gutiérrez, 2010). Possible explanations could be related to the fact that public school children have lower socio-economic status and this could be related to social determinants of violence associated with poverty (e.g., parenting styles, lower level of parental achievement) (Due et al., 2009; Jansen et al., 2012; Tippett and Wolke, 2014; Knaappila et al., 2018).

At the Latin American level, a study that included five countries by 2016, bullying victimization in students aged 13–15 years occurred in at least 37.8% of these, with a higher number of cases in the countries of Peru (47.8%), Bolivia (31.6%), and Honduras (31.6%) (Romo and Kelvin, 2016). Similarly, another study conducted in 16 countries in Latin America reported that just over half of sixth grade schoolchildren reported having suffered violence between peers, with psychological/verbal aggression being more frequent in Argentina (37.18%) and Peru (34.39%), while physical violence was more frequent in Argentina (23.45%) and Ecuador (21.91%) (Román and Murillo, 2011). On the other hand, a study carried out in Brazil reported that 19.8% of students practiced bullying, being more frequent in men (24.2%) (Silva et al., 2019). Evidence of cyberbullying and sexual violence is scarce in Latin American countries (Eljach, 2011; Herrera-López et al., 2018), unlike countries from other continents. Despite the fact that studies conducted in Latin America report a high prevalence of school violence and bullying among students (Román and Murillo, 2011; Herrera-López et al., 2018), there has been little exploration of the types involved. In this context, the situation in Peru is not different from the other countries in the region. According to the available data, school violence reaches a level of 56.4% and bullying 47.5%. However, much of this information is outdated and does not always occupy a consistent terminology to separate the phenomena of school violence and bullying (Oliveros and Barrientos, 2007; Oliveros et al., 2008, 2009; Amemiya et al., 2009; Ministerio de Salud, 2010; Romaní and Gutiérrez, 2010; Romaní et al., 2011).

Peru has certain cultural and geographical particularities that are important to consider when studying phenomena such as school violence and bullying. On the one hand, the Jungle and Sierra region has native communities and indigenous peoples, most of which are rural and still maintain their own culture and customs, while the Coastal region has larger urban areas and greater access to technologies (Instituto Nacional de Estadística e Informática del Perú, 2018a; Instituto Nacional de Estadística e Informática, 2018b). For example, one study collected testimonies from adolescents in the jungle, where they say that sexual exploitation is taken as a normal fact and for this reason they do not denounce it (Mujica et al., 2013). Similarly, in the Peruvian highlands, they do not expect justice from the State and prefer to retaliate against the aggressor themselves, e.g., rondas campesinas (organized groups within the community that impose justice with their own means) (Piccoli, 2009). For example, there are media outlets which report on campesino rondas punishing the alleged rapist or whoever carries out a criminal act (Diaz, 2018; Ticona, 2018). Preliminary studies with small samples, for example, in which three schools in each region of Peru participated, mention that Cusco, which belongs to the highlands region, reported greater psychological and physical violence in children and adolescents, while sexual violence was more prevalent among adolescents in the city of Iquitos, which belongs to the jungle region (Bardales and Huallpa, 2005).

Given this situation, in 2013, the Peruvian government instituted the Specialized System against School Violence (Sistema Especializado contra la Violencia Escolar—SíseVe) to identify and treat cases of school violence and bullying arising within public and private school environments. Systems similar to those in Peru have been developed in other countries. For example, the United States has the SafeSCHOOLS System, in which any type of violence is confidentially reported (Vector Solutions, 2019). Although a review of the literature found a school violence reporting system similar to SíseVe, no articles were found that analyzed and published information reported by this system.

School violence and bullying are social problems within school environments and impede children and adolescents from the basic right to education in safe learning environments. In this sense, it is necessary to determine the proportion of such social problems using consistent and standardized instruments and definitions (Ministerio de Educación, 2014; Cobián-Lezama et al., 2015; Menesini and Salmivalli, 2017). Although there are studies in Peru that have assessed different types of violence, few have assessed the type of aggressor who perpetrated the violence or the type of violence in school settings, and in particular sexual violence at the international level (United Nations Educational Scientific and Cultural Organization [UNESCO], 2019). The majority of available studies have been cross-sectional. with no repetitions over time and have not evaluated all different departments of Peru to establish geographic comparisons (García et al., 2010; Oliveros et al., 2012; Amemiya et al., 2013).

Considering the knowledge gaps found, the objective of this study was to determine the prevalence and prevalence rates of aggressive behaviors according to the different types of school violence and bullying, sociodemographic characteristics, and variation by department, region, and time for the 2014–2018 period in Peru.

## Materials and Methods

### Study Design

This was a cross-sectional descriptive study based on a secondary analysis of the data from SíseVe of the Ministry of Education (Ministerio de Educación—MINEDU) for the period 2014–2018. SíseVe was created in 2013 to record information about school violence cases and bullying perpetrated in school environments of Basic Regular Education [Educación Básica Regular (EBR)] in Peru (Ministerio de Educación, 2014).

### Population and Sample

The target population was the cases reported in SíseVe at the national level, from the opening date, September 2013, until January 2019, obtaining a total of 26,403 reports of school violence in school environments of EBR. For the present secondary analysis, the cases reported within the period January 2014–December 2018, considering 26,078 reports recorded, were evaluated. Cases reporting any type of school violence were included in the study, and cases that did not provide age (*n* = 2,437, 9.3% of the sample considered) were excluded; therefore, the final sample was 23,641 reports (see Supplementary Figure 1).

To obtain the net prevalence rates for types of school violence and bullying, the following formula was used:

$$\mathrm{PREVALENCE}\,\mathrm{RATE}=\frac{\mathrm{Number}\,\mathrm{of}\,\mathrm{existing}\,\mathrm{cases}\,\mathrm{at}\,\mathrm{site}\,X\,\mathrm{and}\,\mathrm{moment}\,\mathrm{in}\,\mathrm{time}}{\mathrm{Total}\,\mathrm{number}\,\mathrm{of}\,\mathrm{persons}\,\mathrm{from}\,\mathrm{the}\,\mathrm{population}\,\mathrm{at}\,\mathrm{the}\,\mathrm{same}\,\mathrm{place}\,\mathrm{and}\,\mathrm{time}}\times 10^{n}$$

The *numerator* corresponds to the number of cases of violence or bullying, the *denominator* is the population of students enrolled during each study period, and the *quotient* obtained was multiplied by 100,000 students (Gordis, 2014). The population of students enrolled for each year of study (2014–2018) was obtained from the Educational Quality of the Ministry of Education (Ministerio de Educación, 2019a).

### Instrument

MINEDU, through its national strategy against school violence, “Paz Escolar” (School Peace), conducted specialized literature reviews, systematic reviews of effective interventions, working meetings with students and government representatives, and consultations in national and international forums to generate an instrument (Ministerio de Educación, 2014) for reporting school violence through an open access platform, SíseVe^1^. The instrument includes questions regarding the characteristics of school violence, characteristics of the victim and aggressor(s), types of violence, frequency of the aggression, reasons for the aggression, and the institution or school to which the victim belongs (Ministerio de Educación, 2018a) (see Supplementary Figure 2).

### Procedure

To promote in order the reporting of violence by the SíseVe platform, MINEDU carries out awareness campaigns advertised through local and national media that finally end up in the schools generating activities with the students using different methodologies, from the promotion of answering the platform to group sessions in which this problem is reflected (Ministerio de Educación, 2019b). Activities are also carried out for the directors and teachers through workshops and sessions on school violence prevention (Ministerio de Educación, 2016); and for the community in general through materials within the virtual platform. The process of reporting cases of violence is presented in two steps: (i) *a personal account*, consisting of the entry of personal data of the person who observed the violence (e.g., director of the school, family, non-family) and/or the victim of aggression; or (ii) a *case report*, which consists of filling out personal data of the victim and data from the school, in addition to questions related to the type of violence. At the end of the process, the person who reported the case receives a list of institutions where they can find help, suggestions, or practical recommendations to deal with what happened and an identification code. The code serves to monitor the case through local, regional, and national authorities (Regional Directorates of Education [Direcciones regionales de Educación (DRE)], Local Educational Management Units [Unidad de Gestión Educativa Local (UGELs) and school environments of EBR] (Ministerio de Educación, 2017, 2018a); the entire procedure mentioned above is available for public scrutiny through a manual on the SiseVe website^2^.

### Variables

The primary variables were *school violence* and *bullying.* The definition and classification were obtained from the United Nations Educational, Scientific and Cultural Organization (UNESCO). *School violence* was classified into three types: Physical (PV), defined as physical attacks, physical fights and corporal punishment, ever perpetrated in the last 30 days; Psychological/Verbal (P/VV), corresponds to verbal, emotional, and social abuse, ever perpetrated in the last 30 days; and Sexual (SV), defined as complete acts, non-consensual sexual attempts, and unwelcome contact perpetrated sometime in the past 365 days. The variables had a dichotomous measurement scale (0 = No, 1 = Yes).

*Bullying* was considered to be harassment committed two or more times perpetrated only by a student or a group of students. Three types of bullying were explored: Physical (PB), defined as hitting, kicking, shoving, forced to do things, perpetrated two or more times in the last 30 days; Psychological/Verbal (P/VB) to verbal, psychological, and social exclusion abuse performed in the last 30 days; and Cybernetic (CB), defined as harassment by text messages and through social networks, publication of unauthorized photographs, emails, and calls in the last 30 days. Sexual bullying is not considered given that the definition and time used by SíseVe (one or more than six times during the year), was not similar to that established by UNESCO (one or more times during the month), document cited in this studio (United Nations Educational Scientific and Cultural Organization [UNESCO], 2019). The variables generated had a dichotomous measurement scale (0 = No; 1 = Yes).

The following *sociodemographic variables* were included: age (years completed), gender (female and male), education level (initial level, primary level, and secondary level), type of school (private and public), departments in Peru (the 24 departments were coded based on the variable Regional Directorates of Education), and region (Costa, Sierra, and Selva). The following *characteristics of aggression* were included*:* type of aggressor (student and staff of schools, the latter includes director, teacher, assistant, or support staff).

### Statistical Analysis

A descriptive analysis was carried out using cross-tabulations for the types of school violence and bullying with the years of study, and prevalence rates were reported. In addition, the types of violence and bullying were crossed with sociodemographic variables (age, gender, and education level), and characteristics of aggression, frequencies/percentages, or average/standard deviation were reported, as appropriate. The departments and regions of Peru also intersected with the main variables and were analyzed to obtain prevalence rates. Finally, statistical graphs were generated reporting the variation in the prevalence rates of the types of school violence and bullying reported in 2018 compared to 2014 to determine trends, according to departments in Peru. The net prevalence rates were calculated per 100,000 enrolled students. Data processing and analysis were performed in the statistical software Stata 15.0 (StataCorp, 2017).

### Ethics Statement

Given that the present study is a secondary data analysis, there was no direct contact with the participants, and the cases were identified by codes; therefore, the possible risks were minimal. However, a commitment was made to the proper use of the information provided by the General Directorate for the Quality of School Management of MINEDU. Additionally, this project was reviewed and approved by the Ethics Committee of the Universidad Católica Los Ángeles de Chimbote (Los Angeles de Chimbote Catholic University), which issued the following report N°003-2019-CIEI-VI-ULADECH-Católica. This project was registered in Open Science Framework at DOI: 10.17605/OSF.IO/TYKF4.

## Results

### Prevalence Rates of Types of School Violence and Bullying According to Reporting Year

The prevalence rates of school violence and general bullying were highest in 2018, 139.2 and 32.5 per 100,000 students, respectively. In relation to the types of school violence and bullying according to the year of reporting, the highest prevalence rate occurred in 2018, with 61.9 cases of psychological/verbal violence and 18.8 cases of psychological/verbal bullying per 100,000 students. When comparing in period 2014–2018 the prevalence rates for the type of violence and psychological/verbal bullying, there was an increase of 45.8 and 11.8 cases per 100,000 students, respectively (Table 1).

**TABLE 1:** Rates of violence and bullying in the 2014–2018 period, according to reporting year.

	2014	2015	2016	2017	2018
**Violence at school**					
Physical	13.5	24.6	34.0	35.8	59.5
Psychological/Verbal	16.1	27.7	39.6	40.5	61.9
Sexual	2.4	4.5	9.0	9.2	17.8
**Bullying**					
Physical	4.5	5.0	7.7	8.9	11.8
Psychological/Verbal	7.0	9.6	13.7	13.5	18.8
Cyberbullying	0.6	0.9	1.3	1.3	1.9

*The rates were calculated per 100,000 students enrolled in EBR.*

### Types of School Violence and Bullying According to Sociodemographic and Aggression Characteristics

Regarding *school violence*, the average age of school children was higher for student victims of sexual violence (12.2 years, SD = 3.4), while the average age was lower for victims of physical violence (10.8 years, SD = 3.8). Sexual and psychological/verbal violence was predominated in females (82 and 50.1%, respectively), and physical violence was more prevalent in males (64.9%). Public institutions were the schools where physical, psychological/verbal, and sexual violence predominated, 84.6, 81.5, and 88.2%, respectively. Aggressors were mostly students of the majority of physical violence (66.4%) and psychological/verbal violence (50.8%), while school staff was the main aggressor of sexual violence (67.8%) (Table 2).

**TABLE 2:** Types of bullying and violence according to sociodemographic and aggression characteristics in the 2014–2018 period.

Variables	School violence			Bullying		
		
	FV	P/VV	SV	FB	P/VB	CB
	*n* (%)	*n* (%)	*n* (%)	*n* (%)	*n* (%)	*n* (%)
**Age**						
Mean (SD)	10.8 (3.8)	11.5 (3.8)	12.2 (3.4)	10.4 (3.5)	12.0 (3.2)	13.9(1.9)
**Sex**						
Female	4,543(35.1)	7,199(50.1)	2,727(82.0)	885 (30.3)	2,335(48.4)	335 (73.2)
Male	8,391(64.9)	7,156(49.9)	598 (18.0)	2,034(69.7)	2,493(51.6)	123 (26.8)
**Degree of instruction**						
Initial level	1,398(10.8)	1,224(8.5)	180 (5.4)	234 (8.0)	144 (3.0)	0 (0.0)
Primary level	5,158(39.9)	5,045(35.2)	998 (30.0)	1,443(49.4)	1,659(34.3)	46 (10.0)
Secondary level	6,378(49.3)	8,086(56.3)	2,147(64.6)	1,242(42.6)	3,025(62.7)	412 (90.0)
**Type of school**						
Private	1,978(15.4)	2,659(18.5)	391 (11.8)	696 (23.4)	1,115(23.1)	178 (38.8)
Public	10596 (84.6)	11,696(81.5)	2,934(88.2)	2,223(76.2)	3,713(76.9)	280 (61.2)
**Type of aggression**						
Between students	8,588(66.4)	7,293(50.8)	1,069(32.2)	2,919(100.0)	4,828(100.0)	458 (100.0)
By school staff***	4,346(33.6)	7,062(49.2)	2,256(67.8)	−	−	−

The total sample for each type of school violence (between students or by school staff against students) and bullying (between students) was obtained from the total cases of students who experience violence within the 2014–2018 period. Abbreviations: PB, physical bullying; P/VB, psychological/verbal bullying; CB, cyberbullying; PV, physical violence; P/VV, psychological/verbal violence; SV, sexual violence. ***School staff (director, teacher, assistant, or support staff).

In relation to *bullying*, the average age was higher in student victims of cyberbullying (13.9 years, SD = 1.9). Cyberbullying was predominant among women (73.2%), while physical and psychological/verbal bullying predominated among men (69.7 and 51.6%, respectively). Public institutions were the schools where physical, psychological/verbal, and cyberbullying predominated, 76.2, 76.9, and 61.2%, respectively. The school children were the type of aggressors that perpetrated greater psychological and verbal bullying (Table 2).

### Types of School Violence and Bullying by Departments in Peru

During the period 2014–2018, in relation to *school violence*, the departments of Tacna (PV = 54.5, P/VV = 60.6) and Loreto (PV = 7.9, P/VV = 10.6) presented the highest and lowest prevalence rates of physical and psychological/verbal violence, respectively; while for sexual violence, Amazonas (15.2) and Apurímac (3.9) presented the highest and lowest prevalence rates, respectively. For *bullying*, the departments of Lima (PB = 13.0, P/VB = 20.1) and Loreto (PB = 1.3, P/VB = 1.8) presented the highest and lowest prevalence rates of physical and psychological/verbal, respectively; while for cyberbullying, the highest and lowest prevalence rates were reported by Tacna (2.5) and Madre de Dios (0.0), respectively. The Costa region had the highest prevalence rate of psychological/verbal violence (45.4), and the Selva region (10.1) had the highest prevalence rate of sexual violence (Table 3).

**TABLE 3:** Prevalence rates by types of school violence and bullying according to departments and regions in Peru, this is the average of the all period 2014–2018.

Variables	Violence at school			Bullying		
		
	PV	P/VV	SV	PB	P/VB	CB
**Department**						
Amazonas	28.0	35.3	15.2	4.5	9.4	0.8
Ancash	37.0	35.5	8.4	6.5	11.3	0.7
Apurímac	17.3	19.7	3.9	2.6	5.0	0.2
Arequipa	31.0	40.7	7.5	5.7	10.9	1.1
Ayacucho	20.8	26.6	12.5	2.3	7.4	0.3
Cajamarca	15.6	18.0	8.2	2.4	4.2	0.4
Cusco	25.2	28.1	5.5	3.4	8.6	0.8
Huancavelica	26.5	30.8	10.5	1.5	6.4	0.2
Huánuco	33.7	29.9	11.1	7.7	11.9	0.6
Ica	29.2	33.0	5.9	6.7	11.9	0.9
Junín	34.8	37.7	11.9	4.9	8.7	0.7
La libertad	25.2	27.7	5.6	5.4	7.5	0.7
Lambayeque	29.9	35.5	7.5	5.8	8.5	0.9
Lima^1^	47.6	53.3	9.2	13.0	20.1	2.3
Loreto	7.9	10.6	6.0	1.3	1.8	0.1
Madre de Dios	27.5	33.1	7.8	6.0	6.9	0.0
Moquegua	35.0	42.3	8.3	7.8	15.1	2.0
Pasco	30.2	32.7	4.8	4.5	10.1	1.1
Piura	34.9	35.8	10.4	8.9	15.5	1.1
Puno	17.3	27.4	5.2	2.9	8.4	0.5
San Martín	41.3	33.6	13.6	9.3	12.7	0.6
Tacna	54.5	60.6	10.2	9.4	17.8	2.5
Tumbes	26.5	29.8	6.9	8.7	11.0	0.9
Ucayali	26.1	23.1	9.9	3.6	7.4	0.8
**Region**						
Costa	40.8	45.4	8.6	10.6	16.5	1.8
Sierra	26.2	29.8	8.3	4.2	8.5	0.6
Selva	23.8	23.6	10.1	4.5	7.0	0.4

*The sample for this department comprises Callao, Lima, and Lima provinces. Abbreviations: PB, physical bullying; P/VB, psychological/verbal bullying; CB, cyberbullying; PV, physical violence; P/VV, psychological/verbal violence; SV, sexual violence.*

### Change Between 2014 and 2018 Prevalence Rates of Types of School Violence and Bullying by Departments in Peru

**FIGURE 1:**
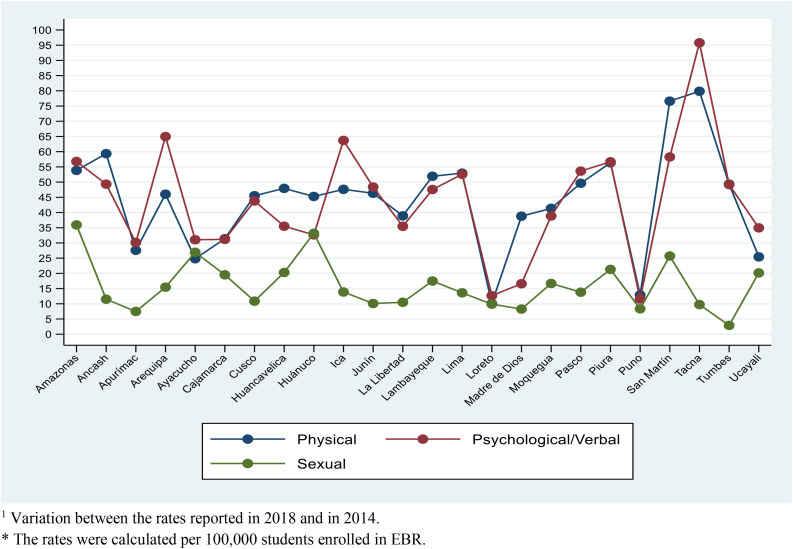
Change in rates reported by type of violence in 2018 compared to 2014, according to departments in Peru. Variation between the rates reported in 2018 and in 2014. The rates were calculated per 100,000 students enrolled in EBR.

The variation in prevalence rates from 2014 to 2018 of psychological/verbal violence by department reported the greatest increase in Tacna (95.8), Arequipa (65.0), and Ica (63.7); for physical violence, the variation rates were highest in Tacna (79.9), San Martín (76.6), and Ancash (59.3); and for sexual violence, the variation rates were highest in Amazonas (35.9), Huánuco (33.2), and Ayacucho (26.9) (Figure 1) (see Supplementary Table 1).

**FIGURE 2:**
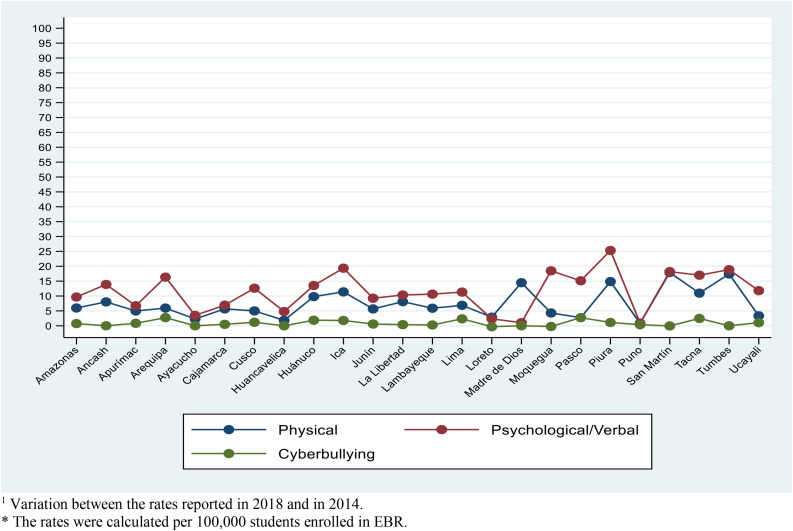
Change in rates reported by bullying type in 2018 compared to 2014, according to departments in Peru. Variation between the rates reported in 2018 and in 2014. The rates were calculated per 100,000 students enrolled in EBR.

The rates of change in prevalence rates from 2014 to 2018 of psychological/verbal bullying by department were higher and increasing in Piura (25.3), Ica (19.4), and Tumbes (18.9). For physical bullying, the variation rates were highest in San Martín (17.9), Tumbes (17.4), and Piura (14.9). Finally, for cyberbullying, the variation rates were highest in Arequipa (2.7), Pasco (2.7), and Tacna (2.5) (Figure 2) (see Supplementary Table 2).

## Discussion

This study is the first to report the prevalence of different types of school violence and bullying by type of aggressor in educational establishments between the period 2014–2018 in Peru. Rates of violence and bullying in all its forms increased during the study period. Sexual violence against women was observed more frequently in public educational institutions, and committed mainly by adult personnel of the educational institution. Finally, we find differences between the coastal, highland, and jungle regions, especially in victimization of sexual abuse in women. Below, we will provide possible explanations of these results.

During the study period, there was an increase in the prevalence rate of cases of violence and bullying in general. For example, the prevalence rate of verbal/psychological violence went from 16.1 in 2014 to 61.9 in 2018 per 100,000 thousand students. Some studies and reports have already shown a high prevalence of verbal violence, reaching 60% of schoolchildren in the regions of Peru (Romaní and Gutiérrez, 2010). Likewise, worldwide estimates of the proportion of children and young people affected by bullying vary specifically between countries and studies, from less than 10% to more than 65% (United Nations Educational Scientific and Cultural Organization [UNESCO], 2011). Among the possible explanations, we must consider that the SíseVe platform has been continuously operating since its implementation and is being disseminated by the Ministry of Education of Peru to support the identification of cases and plan potential interventions. This can produce an effect of increasing knowledge on the part of the educational community, which allows greater access to report cases of perceived violence or bullying. Therefore, the real increase in cases should be considered with caution. However, as has also been observed for other mental health phenomena (Corrigan et al., 2002), as there is greater access to the reporting of this problem, there is a reduction in community stigmatization against these phenomena, which allows greater empowerment to manifest these problems (Saporito et al., 2011). The SíseVe platform for the registration and monitoring of cases of violence and bullying is one of the tactics of the national strategy “School Peace,” created by the Ministry of Education of Peru (Ministerio de Educación, 2014, 2018a,b).

In this study, we found that the highest proportion of sexual violence occurred in female schoolchildren from public educational institutions, and the act was carried out by the staff of the Educational Institution. Our findings are consistent with a study developed in Peru, which found greater sexual violence in adolescent women from public educational institutions (Bardales and Huallpa, 2005). In Latin America, a report from Brazil and Bolivia collected testimonies from schoolchildren who say they have been threatened by their teachers with having sexual relations in exchange for improving their grades and vice versa (Eljach, 2011). Additionally, the UNESCO report carried out worldwide mentions that the prevalence of sexual violence perpetrated by teachers is low (United Nations Educational Scientific and Cultural Organization [UNESCO], 2019). However, a literature review found that in a study conducted in Liberia there was a high prevalence of sexual violence in students perpetrated by teachers and school religious personnel (Steiner et al., 2018), which may account for cultural differences that still exist in relation to the problem of sexual violence. It is important to emphasize the need of studying factors that may be related to sexual violence, as has been the case in our study, where type of aggressor and type of institution or school were included in the analysis. Some authors have expressed concern about the lack of incorporation of these variables given the implications that could occur for the functioning of the educational establishment (Sánchez and Hidalgo, 2019). However, it is relevant to shed light on these issues to finally generate preventive interventions. Unfortunately, Law 29944 “Teacher reform” that accounts for acts of violence in educational establishments does not specify sanction or monitoring of the teacher who commits the act of violence, often being relocated to another educational institution, without having major consequences. In addition to favoring access to the reporting of these acts of violence as a platform for Síseve, it is also important that regulatory adjustments are made to improve the relevant penalties.

During the study period, the Jungle region reported the highest prevalence rate in sexual violence. The few available studies that have explored this relationship are consistent with our results. For example, a study that used the administrative records (since 2002) of the National Program against Family and Sexual Violence of the Ministry of Women and Social Development of Peru also found a higher frequency of sexual violence in students belonging to the cities of the Jungle (Bardales and Huallpa, 2005). An explanation for this situation could be related to the constant and widespread practice of sexual exploitation that we can find in various parts of the jungle (Peruvian Amazon). This situation seems to be related to economic, gender, age, and cultural inequalities (Mujica et al., 2013; Mujica, 2014). On the other hand, some reports in countries with similar characteristics to the Peruvian Jungle have found that sexual violence toward students is perpetrated mostly by teachers and school religious personnel (Steiner et al., 2018), something that in part it is also supported by our results.

Additionally, we found high prevalence of psychological/verbal and physical violence in the coastal and highlands regions. Other authors have shown that this region is the one with the highest indicators of school violence (physical, verbal aggression, social exclusion, among others) in relation to the rest of the country (Romaní et al., 2011). At the same time, various authors point out that the highlands regions is one of the regions where most of the girls and boys who see their rights violated are concentrated (Carpio, 2010), and show lower academic performance (Cueto, 2007). It is known that school violence has been related to contextual situations of greater poverty and family conflicts (Woodworth et al., 1996). In relation to this last point, lower income families tend to present authoritarian parenting practices with greater frequency, prioritizing physical punishment as behavior correction (Hoff et al., 2002). The social theory of learning offers an explanation of how exposure to patterns of violence in the home can perpetuate violent interactions among students in schools (Bandura, 1978). Both victims and perpetrators were found to have experienced harsher parenting (Lereya et al., 2013) and violence (Menesini et al., 2010).

Given this problem, it is suggested to carry out school intervention programs based on evidence. A meta-analysis study concluded that these programs are generally effective, reaching an average decrease of 20–23% (Farrington and Ttofi, 2009; Kärnä et al., 2011). A large−scale evaluation of the KiVa antibullying program: Grades 4–6. Child development, 82(1), 311–330. Despite the heterogeneity of the effect of the programs, they must be intensive and long-lasting, and implemented with fidelity. Involving parents, as well as the use of disciplinary practices with bullies, creating awareness among students about the role of the whole group, and improving the norms and responses against bullying within the classroom have a high impact and effectiveness (Menesini and Salmivalli, 2017). The inclusion of professionals in psychology or psych-pedagogue is crucial, as well as the generation of anti-bullying policy in schools.

The strengths of this study are the use of standardized definitions by UNESCO and exploring the different types of school violence and bullying using reliable data at the national level from MINEDU. Our main limitation is the cross-sectional design, so the causal relationship cannot be guaranteed and the memory bias limitations of the people who report, we do not have violence measures generated from an independent observation. There are variables that we could not include (family, dynamics and parental monitoring, substance use, family income, and parents’ education).

In conclusion, this study makes it possible to advance in the standardization of certain parameters, in such a way that in Peru they allow comparing data between studies in this country and other contexts. During the study period, we found an increase in the prevalence rates of cases of violence and bullying in general. The highest proportion of sexual violence occurred in female schoolchildren from public educational institutions, and the act was carried out by the staff of the Educational Institution. The jungle region had the highest prevalence rate in sexual violence.

### Implications

Bullying or school violence is a public health problem and has short-, medium-, and long-term implications for current schoolchildren and future Peruvian citizens. Therefore, the high prevalence of this phenomenon in our adolescent schoolchildren is a call for attention to design preventive programs with a multidisciplinary approach that deserves this problem.

## Data Availability Statement

The datasets presented in this study can be found in online repositories. The names of the repository/repositories and accession number(s) can be found here: http://www.siseve.pe/web/.

## Ethics Statement

Given that the present study is a secondary data analysis, there was no direct contact with the participants, and the cases were identified by codes; therefore, the possible risks were minimal. However, a commitment was made to the proper use of the information provided by the General Directorate for the Quality of School Management of MINEDU. Additionally, this was reviewed and evaluated by the Ethics Committee of the Universidad Católica Los Ángeles de Chimbote (Los Angeles de Chimbote Catholic University), which issued the following report N°003-2019-CIEI-VI-ULADECH-Católica. Written informed consent from the participants’ legal guardian/next of kin was not required to participate in this study in accordance with the national legislation and the institutional requirements.

## Author Contributions

WA-I developed the original idea and prepared the first draft of the manuscript. WA-I, MI-Z, JB-P, NQ-C, and JG participated in the design of the study. MI-Z executed the statistical analyses. All authors contributed to editing and approving the final version.

## Conflict of Interest

The authors declare that the research was conducted in the absence of any commercial or financial relationships that could be construed as a potential conflict of interest.

## Footnotes

1 http://www.siseve.pe/web/

2 http://www.siseve.pe/web/file/materiales/Orientaciones/Manual_Usuario_Publico.pdf
