# Supplementary material for: Violence at School and Bullying in School Environments in Peru: Analysis of a Virtual Platform
Source: Front Psychol. 2021 Jan 13;11:543991. doi: 10.3389/fpsyg.2020.543991 (PMC7839930; doi:10.3389/fpsyg.2020.543991)
Supplement: Supplementary file 1 [file Data_Sheet_1.docx]

Supplementary Material

## Supplementary Figures

**Supplementary Figure 1**. Flowchart of violence and bullying practised in Peruvian schools: analysis of a virtual platform.

Cases outside the study period

**2013** = 207 cases

**2019**= 118 cases

**Total cases (2013- 2019)**

26403 cases

Missing Data

**Age** = 2437 cases
