# Supplementary material for: Violence at School and Bullying in School Environments in Peru: Analysis of a Virtual Platform
Source: Front Psychol. 2021 Jan 13;11:543991. doi: 10.3389/fpsyg.2020.543991 (PMC7839930; doi:10.3389/fpsyg.2020.543991)

Supplementary Material

**Supplementary Figure 2.** Case report sheet for the Specialized System for Reporting Cases of School Violence (SisseVe).


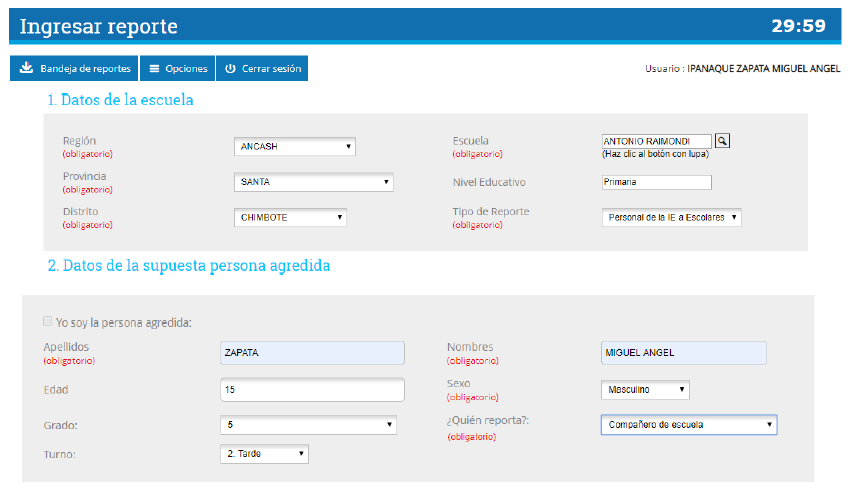


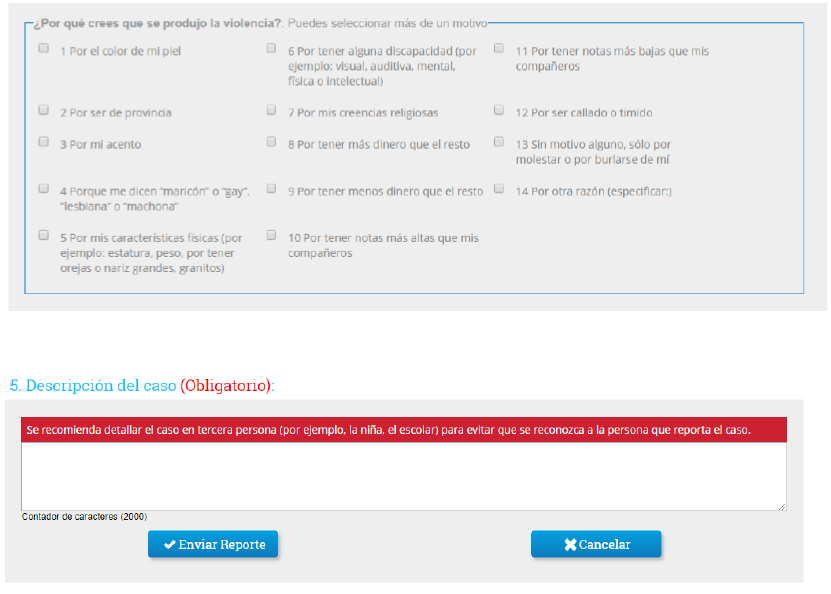

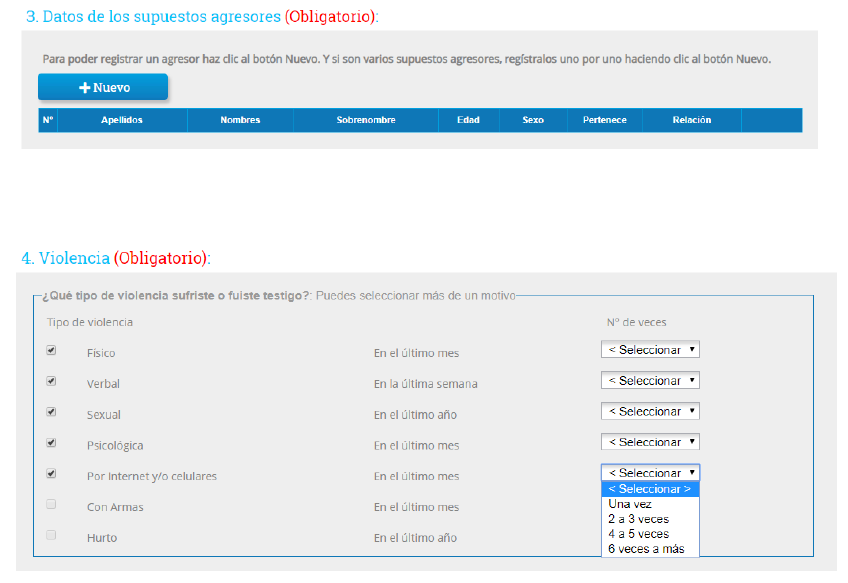

Supplement: Supplementary file 2 [file Data_Sheet_2.docx]
