# Supplementary material for: Violence at School and Bullying in School Environments in Peru: Analysis of a Virtual Platform
Source: Front Psychol. 2021 Jan 13;11:543991. doi: 10.3389/fpsyg.2020.543991 (PMC7839930; doi:10.3389/fpsyg.2020.543991)
Supplement: Supplementary file 3 [file Table_1.docx]

Supplementary Material

# Supplementary Tables

**Supplementary Table 1.** Reported net rates of types of school violence according to departments in Peru for the 2014-2018 period.

|  | **Physical** | | | **Psychological/verbal** | | | **Sexual** | | |
| --- | --- | --- | --- | --- | --- | --- | --- | --- | --- |
|  | **2014** | **2018** | **Difference^1^** | **2014** | **2018** | **Difference^1^** | **2014** | **2018** | **Difference^1^** |
| Amazonas | 0.8 | 54.6 | **53.8** | 2.3 | 59.1 | 56.8 | 0.0 | 35.9 | **35.9** |
| Ancash | 4.5 | 63.8 | **59.3** | 5.8 | 55.1 | 49.3 | 3.1 | 14.6 | **11.5** |
| Apurímac | 3.1 | 30.7 | **27.6** | 4.8 | 34.9 | 30.1 | 0.0 | 7.5 | **7.5** |
| Arequipa | 6.3 | 52.3 | **46.0** | 8.9 | 73.9 | 65.0 | 0.6 | 16.1 | **15.5** |
| Ayacucho | 5.4 | 30.2 | **24.8** | 7.6 | 38.6 | 31.0 | 2.2 | 29.1 | **26.9** |
| Cajamarca | 1.5 | 32.9 | **31.4** | 3.2 | 34.3 | 31.2 | 0.5 | 20.0 | **19.5** |
| Cusco | 2.3 | 47.8 | **45.5** | 3.4 | 47.2 | 43.8 | 0.5 | 11.4 | **10.9** |
| Huancavelica | 9.5 | 57.4 | **47.9** | 17.3 | 52.8 | 35.5 | 1.6 | 21.9 | **20.3** |
| Huánuco | 15.6 | 60.7 | **45.3** | 14.5 | 47.2 | 32.7 | 1.4 | 34.6 | **33.2** |
| Ica | 7.4 | 55.0 | **47.6** | 7.9 | 71.6 | 63.7 | 0.0 | 13.9 | **13.9** |
| Junín | 9.7 | 56.0 | **46.3** | 12.1 | 60.5 | 48.4 | 8.5 | 18.6 | **10.1** |
| La Libertad | 7.9 | 46.8 | **38.9** | 12.1 | 47.6 | 35.5 | 1.4 | 11.9 | **10.5** |
| Lambayeque | 6.8 | 58.7 | **51.9** | 10.2 | 57.8 | 47.6 | 1.0 | 18.5 | **17.5** |
| Lima | 30.5 | 83.4 | **52.9** | 34.8 | 87.4 | 52.6 | 4.1 | 17.7 | **13.6** |
| Loreto | 1.9 | 12.0 | **10.1** | 2.8 | 15.5 | 12.7 | 0.6 | 10.5 | **9.9** |
| Madre de Dios | 2.6 | 41.4 | **38.8** | 10.3 | 26.9 | 16.6 | 0.0 | 8.3 | **8.3** |
| Moquegua | 32.5 | 73.9 | **41.4** | 35.0 | 73.9 | 38.9 | 0.0 | 16.7 | **16.7** |
| Pasco | 8.5 | 58.1 | **49.6** | 15.6 | 69.2 | 53.6 | 1.4 | 15.2 | **13.8** |
| Piura | 4.2 | 60.6 | **56.4** | 2.0 | 58.7 | 56.7 | 1.6 | 22.9 | **21.3** |
| Puno | 8.7 | 21.6 | **12.9** | 14.1 | 25.7 | 11.6 | 1.7 | 10.0 | **8.3** |
| San Martín | 1.4 | 78.0 | **76.6** | 1.8 | 60.1 | 58.3 | 1.3 | 27.0 | **25.7** |
| Tacna | 9.1 | 89.0 | **79.9** | 11.8 | 107.6 | 95.8 | 2.7 | 12.4 | **9.7** |
| Tumbes | 0.0 | 49.3 | **49.3** | 3.0 | 52.2 | 49.2 | 0.0 | 2.9 | **2.9** |
| Ucayali | 7.5 | 32.9 | **25.4** | 5.4 | 40.4 | 35.0 | 4.0 | 24.2 | **20.2** |

^1^ Variation between the rates reported in 2018 and in 2014.

* The rates were calculated per 100,000 students enrolled in EBR.
