# Supplementary material for: Violence at School and Bullying in School Environments in Peru: Analysis of a Virtual Platform
Source: Front Psychol. 2021 Jan 13;11:543991. doi: 10.3389/fpsyg.2020.543991 (PMC7839930; doi:10.3389/fpsyg.2020.543991)
Supplement: Supplementary file 4 [file Table_2.docx]

Supplementary Material

**Supplementary Table 2.** Net rates reported by type of bullying according to departments in Peru for the 2014-2018 period.

|  | **Physical** | | | **Psychological/verbal** | | | **Cyberbullying** | | |
| --- | --- | --- | --- | --- | --- | --- | --- | --- | --- |
|  | **2014** | **2018** | **Difference^1^** | **2014** | **2018** | **Difference^1^** | **2014** | **2018** | **Difference^1^** |
| Amazonas | 0.0 | 6.0 | **6.0** | 1.6 | 11.2 | **9.7** | 0.0 | 0.7 | **0.7** |
| Ancash | 2.7 | 10.7 | **8.0** | 2.0 | 15.9 | **13.9** | 0.7 | 0.7 | **0.0** |
| Apurímac | 0.0 | 5.0 | **5.0** | 1.6 | 8.3 | **6.7** | 0.0 | 0.8 | **0.8** |
| Arequipa | 1.6 | 7.6 | **6.0** | 1.0 | 17.3 | **16.3** | 0.0 | 2.7 | **2.7** |
| Ayacucho | 1.1 | 3.4 | **2.3** | 3.8 | 7.3 | **3.5** | 0.0 | 0.0 | **0.0** |
| Cajamarca | 0.0 | 5.7 | **5.7** | 0.5 | 7.4 | **6.9** | 0.0 | 0.5 | **0.5** |
| Cusco | 0.6 | 5.6 | **5.0** | 0.9 | 13.5 | **12.6** | 0.3 | 1.5 | **1.2** |
| Huancavelica | 0.0 | 1.8 | **1.8** | 1.6 | 6.4 | **4.8** | 0.0 | 0.0 | **0.0** |
| Huánuco | 2.3 | 12.1 | **9.8** | 5.2 | 18.7 | **13.5** | 0.0 | 1.9 | **1.9** |
| Ica | 2.5 | 13.9 | **11.4** | 3.5 | 22.8 | **19.4** | 0.0 | 1.8 | **1.8** |
| Junín | 2.4 | 8.1 | **5.7** | 3.3 | 12.6 | **9.3** | 0.0 | 0.6 | **0.6** |
| La Libertad | 2.5 | 10.6 | **8.1** | 3.8 | 14.2 | **10.4** | 0.7 | 1.1 | **0.4** |
| Lambayeque | 2.7 | 8.6 | **5.9** | 3.4 | 14.0 | **10.6** | 0.7 | 1.0 | **0.3** |
| Lima | 11.0 | 17.9 | **6.9** | 17.5 | 28.8 | **11.3** | 1.3 | 3.6 | **2.3** |
| Loreto | 0.3 | 3.2 | **2.9** | 0.0 | 2.3 | **2.3** | 0.3 | 0.0 | **-0.3** |
| Madre de Dios | 0.0 | 14.5 | **14.5** | 5.1 | 6.2 | **1.1** | 0.0 | 0.0 | **0.0** |
| Moquegua | 10.0 | 14.3 | **4.3** | 12.5 | 31.0 | **18.5** | 5.0 | 4.8 | **-0.2** |
| Pasco | 1.5 | 4.2 | **2.7** | 4.3 | 19.4 | **15.1** | 1.5 | 4.2 | **2.7** |
| Piura | 1.6 | 16.5 | **14.9** | 1.0 | 26.3 | **25.3** | 0.2 | 1.3 | **1.1** |
| Puno | 2.3 | 3.1 | **0.8** | 3.6 | 4.5 | **0.9** | 0.3 | 0.7 | **0.4** |
| San Martín | 0.0 | 17.9 | **17.9** | 0.9 | 19.1 | **18.2** | 0.4 | 0.3 | **-0.1** |
| Tacna | 2.6 | 13.6 | **11.0** | 5.3 | 22.3 | **17.0** | 0.0 | 2.5 | **2.5** |
| Tumbes | 0.0 | 17.4 | **17.4** | 0.0 | 18.9 | **18.9** | 0.0 | 0.0 | **0.0** |
| Ucayali | 0.6 | 4.0 | **3.4** | 2.0 | 13.8 | **11.8** | 0.6 | 1.7 | **1.1** |

^1^Variation between the rates reported in 2018 and in 2014.

^*^The rates were calculated per 100,000 students enrolled in EBR.
